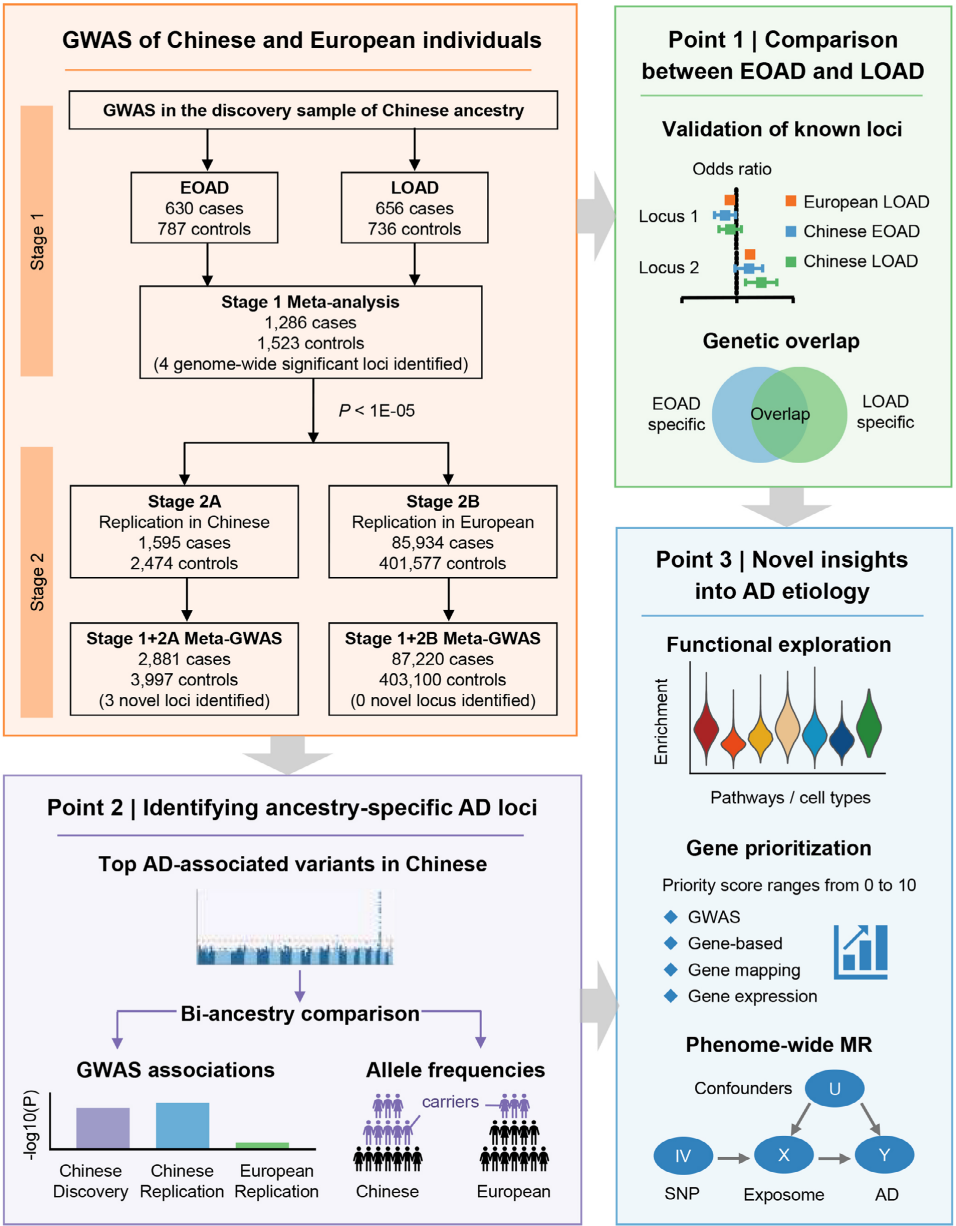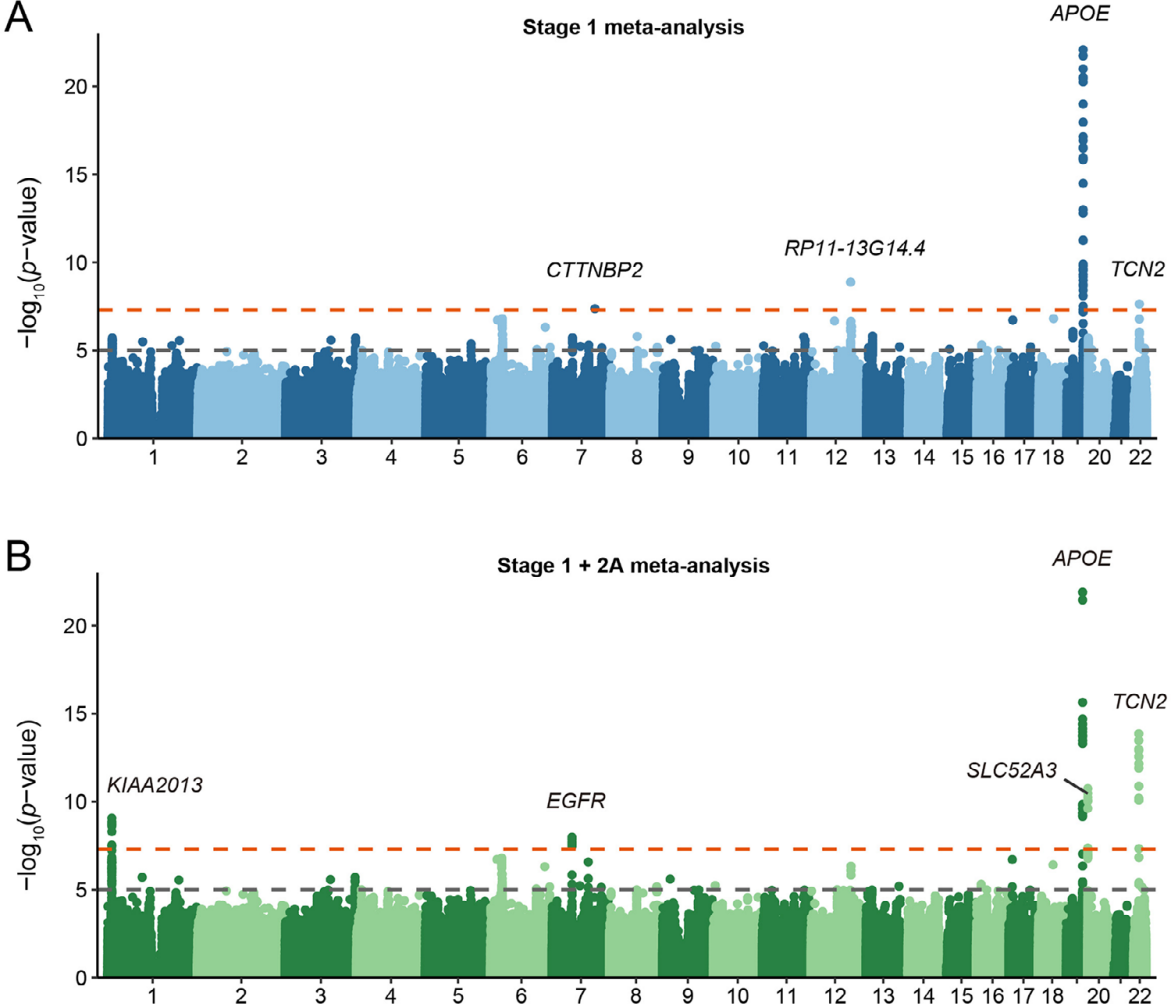# Genome‐wide association meta‐analyses in Chinese and European individuals yield new insights into the etiology of Alzheimer’s disease

**DOI:** 10.1002/alz.084356

**Published:** 2025-01-03

**Authors:** Yi‐Jun Ge, Shi‐Dong Chen, Bang‐Sheng Wu, Ya‐Ru Zhang, Jun Wang, Xiao‐ Yu He, Qianhua Zhao, Yan‐Jiang Wang, Jian‐Ping Jia, Jin‐Tai Yu

**Affiliations:** ^1^ Huashan Hospital, Fudan University, Shanghai China; ^2^ Daping Hospital, Third Military Medical University, Chongqing China; ^3^ Xuanwu Hospital, Capital Medical University, Beijing China; ^4^ Huashan Hospital, Fudan University, Shanghai, Shanghai China

## Abstract

**Background:**

Alzheimer’s disease (AD) is a devastating neurological disease with complex genetic etiology, yet most known loci were only identified from the late‐onset type of European ancestry.

**Method:**

We performed a two‐stage genome‐wide association study (GWAS) of AD totaling 6,878 Chinese and 487,511 European individuals.

**Result:**

We demonstrated a shared genetic architecture between early‐ and late‐onset AD. In addition to the APOE locus, our GWAS of two independent Chinese samples uncovered three novel AD susceptibility loci (KIAA2013, SLC52A3, and TCN2) and a novel ancestry‐specific variant within EGFR (rs1815157). Notably, the TCN2 locus showed genome‐wide significant associations with AD in both discovery and replication stages in Chinese populations. More replicated variants were observed in the Chinese (31%) than European samples (4%). Combining genome‐wide associations and functional annotations, EGFR and TCN2 were prioritized as two of the most biologically significant genes. Phenome‐wide Mendelian randomization suggests that high mean corpuscular hemoglobin concentration might be protective against AD.

**Conclusion:**

The current study reveals novel AD susceptibility loci, emphasizes the importance of diverse populations in AD genetic research, and advances our understanding of disease etiology.